# Hordatines and Associated Precursors Dominate Metabolite Profiles of Barley (*Hordeum vulgare* L.) Seedlings: A Metabolomics Study of Five Cultivars

**DOI:** 10.3390/metabo12040310

**Published:** 2022-03-31

**Authors:** Claude Y. Hamany Djande, Paul A. Steenkamp, Lizelle A. Piater, Fidele Tugizimana, Ian A. Dubery

**Affiliations:** Research Centre for Plant Metabolomics, Department of Biochemistry, University of Johannesburg, P.O. Box 524, Auckland Park, Johannesburg 2006, South Africa; 201410297@student.uj.ac.za (C.Y.H.D.); psteenkamp@uj.ac.za (P.A.S.); lpiater@uj.ac.za (L.A.P.); ftugizimana@uj.ac.za (F.T.)

**Keywords:** anti-microbial metabolites, barley, *Hordeum vulgare*, liquid chromatography, mass spectrometry, metabolomics, multivariate data analysis, secondary metabolites

## Abstract

In the process of enhancing crop potential, metabolomics offers a unique opportunity to biochemically describe plant metabolism and to elucidate metabolite profiles that govern specific phenotypic characteristics. In this study we report an untargeted metabolomic profiling of shoots and roots of barley seedlings performed to reveal the chemical makeup therein at an early growth stage. The study was conducted on five cultivars of barley: ‘Overture’, ‘Cristalia’, ‘Deveron’, ‘LE7′ and ‘Genie’. Seedlings were grown for 16 days post germination under identical controlled conditions, and methanolic extracts were analysed on an ultra-high performance liquid chromatography coupled to high-resolution mass spectrometry (UHPLC–HRMS) system. In addition, an unsupervised pattern identification technique, principal component analysis (PCA), was performed to process the generated multidimensional data. Following annotation of specific metabolites, several classes were revealed, among which phenolic acids represented the largest group in extracts from both shoot and root tissues. Interestingly, hordatines, barley-specific metabolites, were not found in the root tissue. In addition, metabolomic profiling revealed metabolites potentially associated with the plants’ natural protection system against potential pathogens. The study sheds light on the chemical composition of barley at a young developmental stage and the information gathered could be useful in plant research and biomarker-based breeding programs.

## 1. Introduction

Barley is a small grain crop belonging to the Triticeae tribe in the grass family of Poaceae (Gramineae) [1,2]. Among the *Hordeum* genus, cultivated barley (*H. vulgare* L.) and its wild progenitor (*Hordeum spontaneum* C. Koch) are the most reported [3,4]. The crop can thrive in a wide range of climates around the world and is mainly employed as a substrate for malting in the brewing industry. Approximately 90% of the world’s malt production is generated from barley, owing to its enzymatic and husk properties [5,6,7]. Although barley is not commonly used as food, its regular consumption is associated with multiple health benefits. Barley is also considered a model in plant research, especially when investigating environmental stress resistance. In terms of metabolite diversity and content, barley is comparable to other major cereals grains [6,8].

The plant possesses some distinctive phytochemical components, including all eight tocol vitamins (bioactive compounds with anti-oxidant traits) [6,8]. The anti-oxidant group also contains phenolamides, also known as cinnamamides or phenylamides or hydroxycinnamic acid amides (HCAAs). These are a group of phenolic compounds resulting from the conjugation of aliphatic or aromatic (poly)amines (e.g., agmatine, spermidine, putrescine and spermine) with phenolic moieties, principally hydroxycinnamic acids (HCAs: caffeic, *p*-coumaric, ferulic and sinapic acids and derivatives), due to acyltransferase enzymes [9,10,11]. Although widely distributed in the plant kingdom, the biological and physiological functions of HCAAs are still poorly understood. HCAAs have also been reported as potent anti-oxidant, anti-inflammatory, anticancer and anti-microbial compounds [9], and their occurrence has been associated with plant defence against biotic and abiotic stresses [11,12].

**Figure 1:**
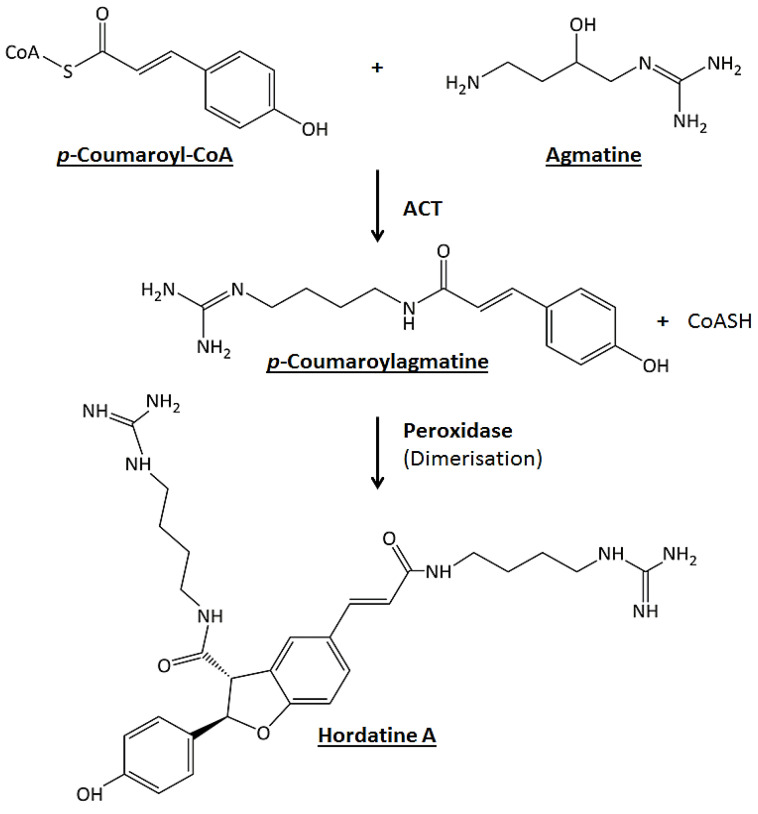
Hordatine A biosynthesis. The first step is a coumaroyltransferase (ACT)-catalysed reaction of *p*-coumaroylCoA and agmatine resulting in the formation of *p*-coumaroylagmatine. The second step is the oxidative coupling of two molecules of *p*-coumaroylagmatine in the presence of peroxidase.

New cultivars with superior and desirable traits are constantly being developed as a result of active breeding initiatives. However, the underlying molecular fingerprints that functionally explain these features are often unknown. Several studies have applied different metabolomics approaches to elucidate the metabolite composition of plant cultivars and to identify important discriminatory biomarkers [13,14]. Metabolite profiling and metabo-phenotyping of cultivars at an early developmental stage can provide rapid insights into the chemical composition of a plant and assist breeders in the selection of desired phenotypic traits that can protect against, e.g., post-germination damping-off diseases. Hordatines are HCAAs typical of barley, naturally occurring during the development of the seedling but also inducible after pathogen attacks. These specialised metabolites have analogous structures and are biosynthesised from the polyamine agmatine and the coenzyme A derivatives of hydroxycinnamic acids (*p*-coumaric, ferulic and sinapic acid) [15,16,17]. Hordatines A and B were the first to be characterised and their biosynthesis involves two enzymes: agmatine coumaroyltransferase (ACT) and peroxidase. The former catalyses the formation of either *p*-coumaroylagmatine or feruloylagmatine from the respective HCA-Coenzyme A with agmatine. The latter catalyses the oxidative coupling of HCAAs to produce the corresponding hordatines (Figure 1) [15]. There is limited information about this class of compounds in general but an even greater lack of information about hordatines C and D. However, it has been assumed that their biosynthesis and function might be similar to those of hordatines A and B. Derivatives of hordatines include methylated, hydroxylated and glycosylated compounds [16,18,19].

Recently, growing interest has been directed at this class of compounds because of their importance in diverse areas [16,20]. For plant science and crop improvement research, the most attractive property of hordatines remains their exceptional anti-fungal properties. In this study, aiming to explore the metabolite composition of the above- and below-ground tissues (shoots vs. roots) of barley, metabolite profiling was carried out on five cultivars at the seedling stage of development. Ultra-high performance liquid chromatography (UHPLC) coupled to mass spectrometry (MS) revealed a metabolite fingerprint broadly common to all cultivars, featuring not only the hordatines but also several metabolite classes with potential anti-microbial properties and potential utilisation as chemical deterrents in plant defence.

## 2. Results and Discussion

### 2.1. Metabolite Profiling of Barley Shoot and Root Tissues

**Figure 2:**
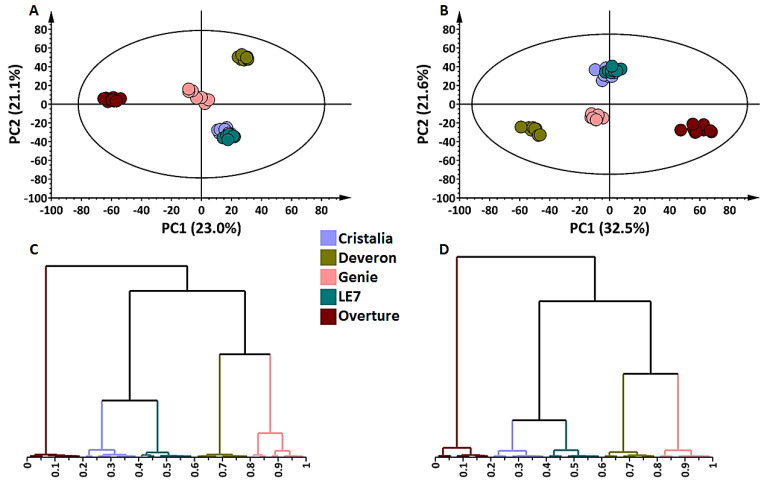
Principal components analysis (PCA) score plot models and hierarchical clustering analyses (HiCA) for shoot and root tissues of five cultivars of *Hordeum vulgare* (Northern Cape region of South Africa). The calculated Hoteling’s T2 with a 95% confidence interval is represented by the ellipses present in each PCA model. (**A**) Shoot tissue: five-component model explaining 63.2% variation (R^2^X*cum*) and predicting 50.7% variation (Q^2^*cum*). (**B**) Root tissue: five-component model explaining 71.8% variation (R^2^X*cum*) and predicting 61.2% variation (Q^2^*cum*). (**C**) HiCA dendrogram showing the hierarchical structure of shoot data and corresponding to the PCA model in (**A**). (**D**) HiCA dendrogram showing the hierarchical structure of root data and corresponding to the PCA model in (**B**). Data were acquired from hydromethanolic extracts and analysed by UHPLC–qTOF-MS in ESI(–) mode.

As previously mentioned, the hyphenated analytical platform UHPLC–qTOF-MS was used in both positive and negative ionisation modes to provide insights into the chemical composition of barley plant extracts. Scrutinising the metabolite composition of the above- and below-ground tissues of the cultivars is important to understand tissue-specific traits thereof. The complexity and diversity of barley metabolites ranged from polar to non-polar, as depicted in the base peak intensity (BPI) chromatograms from the shoot and root tissues (Figures S1 and S2). Due to the high dimensionality of the data, the unsupervised chemometric method, principal component analysis (PCA), was used and generated a summary of general trends in the dataset, illustrating the similarities or differences within (intra-cultivar variance by PC2) and between (inter-cultivar variance by PC1). The PCA score plots of the shoot and root samples in ESI(–) mode revealed evident and similar clustering patterns. All cultivars clustered distantly from each other except for ‘Cristalia’ and ‘LE7′ cultivars which grouped close to each other (Figure 2A,B). In ESI(+) mode, identical observations were made in the shoots but an individual grouping of all five cultivars was noticeable in the roots (Figure S3). Furthermore, the generated hierarchical cluster analysis (HiCA) dendrograms revealed a similar pattern observed in the equivalent PCA graphs; however, additional information could be obtained, such as the closed branching of shoot and root samples from ‘Deveron’ and ‘Genie’ (Figure 2C,D). These findings may be correlated with the genetic background of the cultivars, ‘Cristalia’ being a parent to ‘LE7′ and ‘Genie’ being a parent to ‘Deveron’. Sample grouping or branching is often relative to the metabolome differences or similarities existing therein.

A total of 78 metabolites were putatively annotated, as reported in Table 1. Except for isovitexin 2″-*O*-glucoside and a derivative of citric acid, all annotated metabolites were found in all the cultivars under investigation, suggesting a narrow genetic base shared by these cultivars and experimental lines. The annotated metabolites were amino acids and derivatives, alkaloids, organic acids, fatty acids and phenolic compounds, which included phenolic acids and derivatives as well as flavonoids. Phenolic compounds represented the largest class of metabolites annotated in both tissues. The size of the class was mostly attributed to phenolic acids and more particularly to hydroxybenzoic acids (HBAs), HCAs, HCAAs and benzofurans. Among these, only HBAs and HCAAs were found in roots.

#### 2.1.1. Phenolic Compounds: Phenolic Acids and Derivatives and Flavonoids

Structurally, phenolic compounds contain one or multiple aromatic rings attached to one or more hydroxyl substituents [21]. This group often includes phenolic acids/HCAs, coumarins, tannins, stilbenes and flavonoids. The former and the latter were among the main classes of compounds annotated in the study. As part of phenolic acids, two bound HBAs were annotated in both shoot and root tissues: gallic acid monohydrate (compound **2**) and protocatechuic acid (dihydroxybenzoic acid) hexose (compound **1**) [22,23]. In addition, an intermediate compound, benzylalcohol-hexose pentose (compound **2**), was annotated, as previously described [23]. Similarly to [17], three HCAs, ferulic, caffeic and sinapic acid, were only found in shoot tissue, conjugated either to quinic acid, forming chlorogenic acids (CGAs), or glycosylated to a hexose (compounds **4–8**). The compounds were 3- and 4-feruloylquinic acids, 3-caffeoylquinic acid, ferulic acid hexose and sinapic acid hexose [17,24,25]. Among the phenolic acids, ferulic acid has been reported to be the most abundant in cereals [26,27].

**Figure 3:**
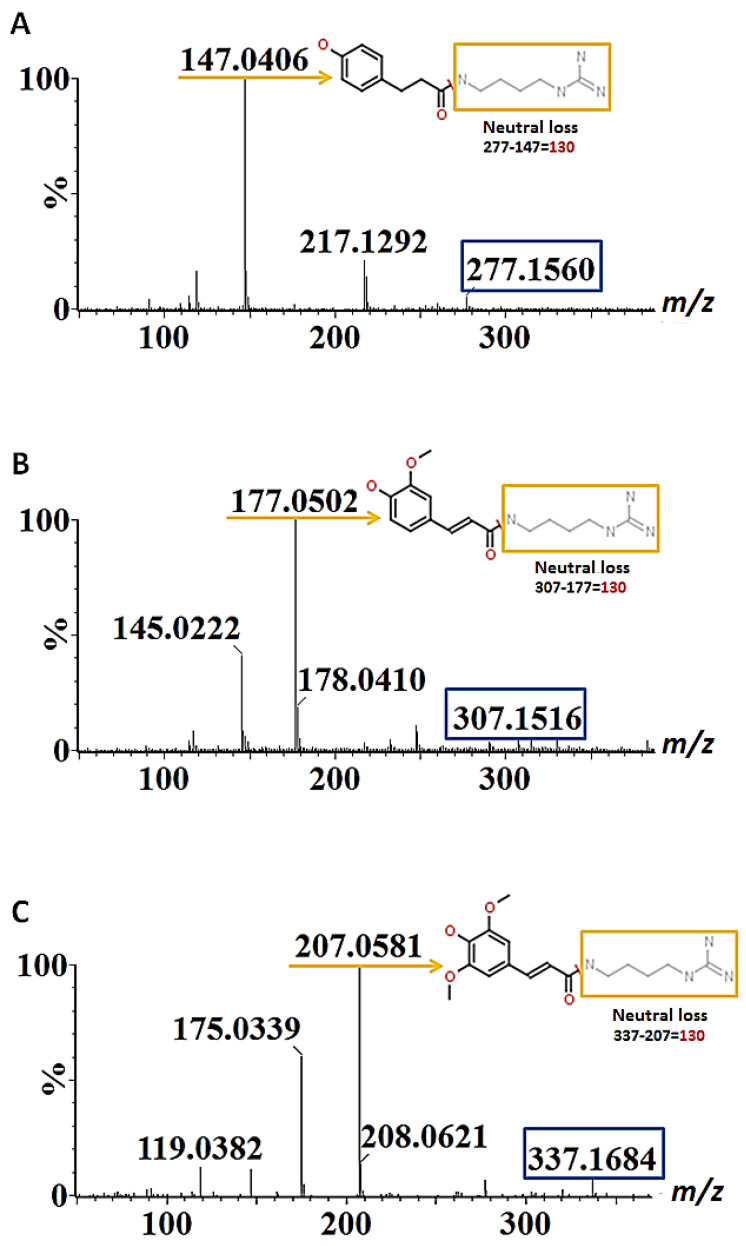
Mass fragmentation patterns of (**A**) *p*-coumaroylagmatine (*m*/*z* 277), (**B**) feruloylagmatine (*m*/*z* 307) and (**C**) sinapoylagmatine (*m*/*z* 337) characterised from barley root samples in the positive ionisation mode. The compounds exhibit identical neutral loss (*m*/*z* 130) and fragments corresponding to their dehydroxylated hydroxycinnamoyl moieties. The blue rectangles indicate the precursor ions, the orange arrows indicate the base peak fragment ions and the neutral loss fragments (*m/z* 130) are indicated in orange rectangles.

Furthermore, thirteen HCAAs (compounds **9–21**) were annotated: *p*-coumaroylputrescine, feruloylagmatine isomer I and two isomers of sinapoylagmatine only found in roots; hexosylated derivatives of feruloylagmatine, feruloylhydroxyagmatine, *p*-coumaroylagmatine and *p*-coumaroylhydroxyagmatine found in the shoots; and, finally, *p*-coumaroylhydroxyagmatine, *p*-coumaroylagmatine, *p*-coumaroylhydroxyagmatine, feruloylhydroxyagmatine, feruloylagmatine isomer II and sinapoylhydroxyagmatine, found in both tissue types. The fragmentation patterns of these compounds were more evident in the ESI(+) ionisation mode and are shown in Figure 3A–C as a representation of spectral information acquired. *p*-Coumaroylagmatine, feruloylagmatine and sinapoylagmatine were characterised by the presence of a dehydroxylated hydroxycinnamoyl moiety (*p*-coumaroyl, feruloyl and sinapoyl) and the neutral loss of 130 corresponding to the molecule agmatine [16,17,18]. In the case of *p*-coumaroylputrescine, the fragment ion *m*/*z* 147 resulted from the neutral loss of *m*/*z* 88 corresponding to the mass of putrescine [28].

**Figure 4:**
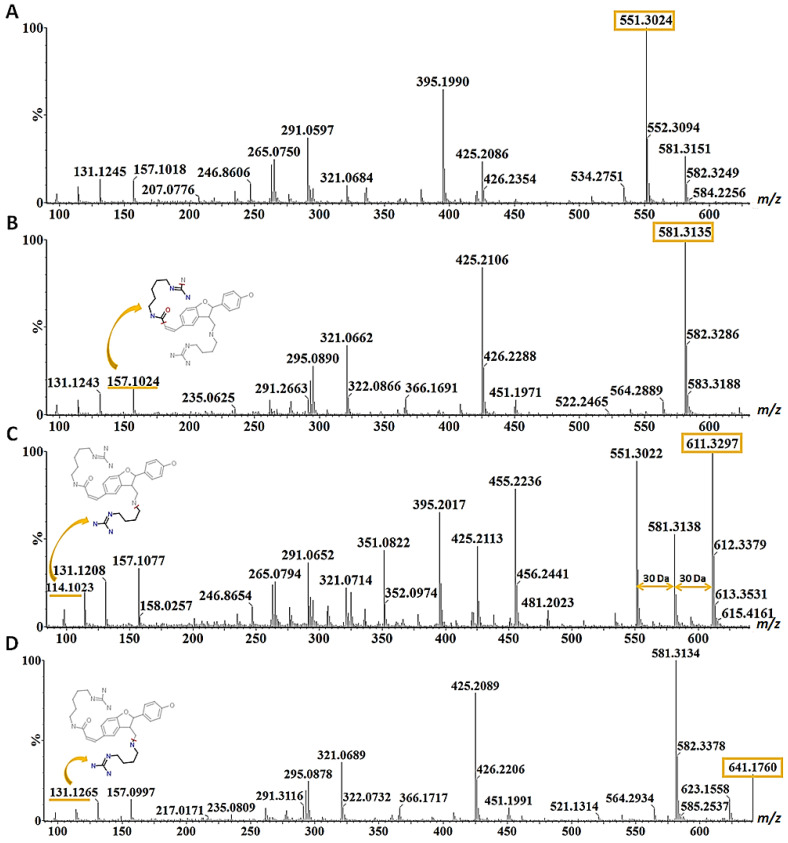
Mass fragmentation patterns of (**A**) hordatine A (*m*/*z* 551), (**B**) hordatine B (*m*/*z* 581), (**C**) hordatine C (*m*/*z* 611) and (**D**) hordatine D (*m*/*z* 641) characterised from barley shoot samples in the negative ionisation mode. The precursor ions are indicated with orange rectangles and the mass difference of 30 among the hordatines is shown by the double arrows. The compounds were all characterised by the presence of ions with *m*/*z* 131, 147 and 157 in each spectrum, and structures of these fragment ions generated on the ‘Massfrag’ tool of the MassLynx software are indicated with the orange arrow.

The dimerisation of HCAAs to generate the hordatines (Figure 1) also occurred and was interestingly observed only in extracts from barley shoots; these were hordatine A, two isomers of hordatine B and hordatine C and D (compounds **22–26**). Chemically, hordatines are described as benzofurans, guanidines, phenols, dicarboxylic acid diamides and aromatic ethers (https://pubchem.ncbi.nlm.nih.gov/compound/Hordatine-A, accessed on 12 January 2022). Hordatine A results from the condensation of two molecules of *p*-coumaroylagmatine; hordatine B, on the other hand, is a mixture of *p*-coumaroylagmatine and feruloylagmatine; hordatine C derives from two molecules of feruloylagmatine; and, lastly, hordatine D consists of sinapoylagmatine and feruloyagmatine [15,16,17,18]. Hordatine A and B displayed a mass difference of 30 Da (581 − 551 = 30) also observed on the precursors (307 − 277 = 30; 337 − 307 = 30) and corresponding to the methoxyl group. Hordatine C and D were characterised by the exhibition of the same mass difference (611 − 581 = 30, 641 − 611 = 30) and also their occurrence in the same cluster with hordatines A and B [16]. All four hordatines clustered closely to each other and the observed poor baseline resolution of peaks did not allow the generation of clear spectra that only featured the compound of interest (Figure 4A–D). A similar observation was made by [18]. However, the presence of diagnostic fragment ions *m*/*z* 157, 131 and 114, all deriving from the agmatine residue, was noted, hence the need to develop a UHPLC method capable of fully discriminating among the hordatines, associated derivatives and different isomers in future research.

**Figure 5:**
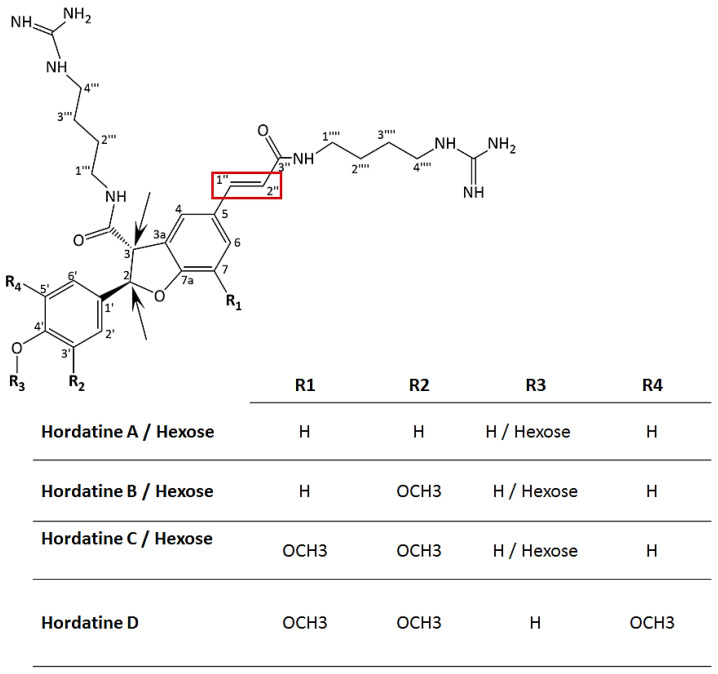
Structures of hordatines A, B, C and D as well as their corresponding glycosylated derivatives. Hordatine D is proposed by analogy with previously reported structures of A, B and C. Two chiral centres existing on carbon 2 and 3 of the dihydrobenzofuran moiety present in the core structures are indicated with black arrows. The red rectangle represents the *cis*/*trans* geometric isomer site.

Considering the huge number of HCA modifications occurring in nature, the *O*-glycosylation of the HCAAs is not unexpected, especially as plants often perform glycosylation of compounds to change their stability, toxicity, hydrophilicity, localisation and bioactivity [29]. Two isomers of hordatine C hexose (compounds **27** and **30**) were annotated, as well as hexose conjugates of hordatine A and hordatine B (compounds **28** and **29**) [16,17]. The isomerisation (*cis* and *trans*) of hordatines was previously proposed [30,31,32] to occur at the two chiral centres existing on carbon 2 and 3 of the dihydrobenzofuran moiety present in their core structure (Figure 5). By analogy with HCAs, another isomerisation site could be proposed to occur at positions 1′’ and 2′’.

In the case of flavonoids, 18 were annotated in the shoot tissue: 14 flavones, a flavanone and a flavonoid-related compound. Flavones included apigenin (compounds **32**, **33**, **35–38**; **40**, **42** and **43**), luteolin (compounds **31**, **39** and **47**), chrysoeriol (3′-methoxy derivative of luteolin; compound **39**) and isoscoparin (compounds **34**, **41**, **44**) substituted with di- and triglycosides by *O*-glycosidic bonds, as well as cinnamic acid moieties (ferulic and sinapic acids) at different positions [17]. The prenylated flavonone, 6-prenylnaringenin (compound **46**) was characterised as previously described [33]. Only one apigenin derivative, saponarin (or isovitexin-7-*O*-glucoside, compound **32**), was annotated in the root tissue. The compound was first reported in root exudates [34].

#### 2.1.2. Fatty Acids and Derivatives

Fatty acids and derivatives represent a major class of the annotated metabolites. These included derivatives of linolenic (18:3) and linoleic (18:2) acids which are two of the most abundant unsaturated fatty acids (UFAs) in plants. As part of UFA derivatives, N-acylethanolamide of α-linolenic and linoleic acids, were identified in root samples as α-linolenoyl and linoleoyl ethanolamide (compounds **59**, **60**) [35]. In addition, hydroxylinoleic acid (compound **72**) [36], three isomers of trihydroxyoctadeca-10,15-dienoic acid (compounds **61**, **62**, **63**), a trihydroxyoctadecenoic acid (compound **64**), 9-oxo-12,13-dihydroxy-10E,15Z-octadecadienoic acid (9K,12,13-diHODE) (compound **71**), two isomers of 9-hydroxy-12-oxo-10,15-octadecadienoic acid (12K,9-HODE; compound **68**) and three unknown derivatives of linolenic acid (compounds **70**, **73** and **78**) were identified and annotated in the current study. The biosynthetic precursor of jasmonic acid, 12-oxo-phytodienoic acid (12-OPDA, compound **71**), was also annotated, together with two isomers of a conjugate (compounds **66** and **67**). The two isomers of OPDA (with *m*/*z* 309) were detected by the presence of a fragment, with *m*/*z* 291 corresponding to that of 12-OPDA, and other fragments with *m*/*z* 247 and 165 similar to those observed in the 12-OPDA spectrum [37,38]. In addition, a jasmonic acid derivative, 12-hydroxyjasmonate sulphate (compound **58**), was also found in leaf tissue extracts. Lastly, α-linolenic acid was found associated with a glycerol molecule to form α-linolenoylglycerol or monolinolenin, and four isomers of that compound were annotated in the positive ionisation mode (compounds **74–77**).

#### 2.1.3. Organic Acids, Amino Acids and Alkaloids

The polar organic acids and amino acids listed in Table 1, together with the single alkaloid, accounted for the minority of the annotated metabolite classes. This is due to their polar natures and low extractability into organic solvents. All organic acid compounds annotated here were found in both tissue types, except for a citric acid derivative (compound **57**) only found in shoot tissue. These compounds were citric acid/isocitric acid (compounds **55** and **53**), annotated as described by [39], succinic acid (compound **56**) and malic acid (compound **54**) [40]. A derivative of citric acid (*m*/*z* 306.0423) presenting similar characteristic fragment ions was also identified.

With regards to amino acids, phenylalanine (Phe) (compound **50**) and tryptophan (Trp) (compound **51**) were identified in extracts from both shoot and root tissues. Phe was characterised in the positive ionisation mode by the presence of a fragment with *m*/*z* 120 resulting from the loss of a H_2_O molecule and a CO residue. Another characteristic ion was that of *m*/*z* 103, resulting from the additional loss of an NH_3_ group. This MS pattern was described by [41] as a major fragmentation pathway as opposed to the minor fragmentation one observed in this study in the negative ionisation mode. This minor fragmentation pathway of the deprotonated Phe generated fragments with *m*/*z* 147 as a result of NH_3_ loss, and *m*/*z* 103, resulting from the subsequent loss of a H_2_O molecule and a CO residue. On the other hand, Trp was characterised by the presence of the fragment ion with *m*/*z* 188 subsequent to the dissociation of an NH_3_ group. Following additional losses of CH_2_CO and CO_2_ residues, fragment ions with *m*/*z* 146 and 144, respectively, were generated. The further detachments of CO and subsequently HCN residues from the fragment with *m*/*z* 146 produced additional smaller fragments of *m*/*z* 118 and 91, respectively [41]. Finally, the acylated dipeptide N-acetylaspartylglutamic acid (compound **52**), derived from aspartic and glutamic acids, was only found in the shoot tissue and characterised using the Chemspider, PubChem and DNP databases.

Lastly, hordenine (N,N-dimethyltyramine, compound **49**) was identified at a Rt = 1.17 min and characterised in both tissues by the presence of a parent ion with *m*/*z* 166 and a daughter ion with *m*/*z* 121 (in the positive ionisation mode), resulting from the removal of the amine group.

### 2.2. Pathways Involved in the Biosynthesis of Annotated Metabolites

**Figure 6:**
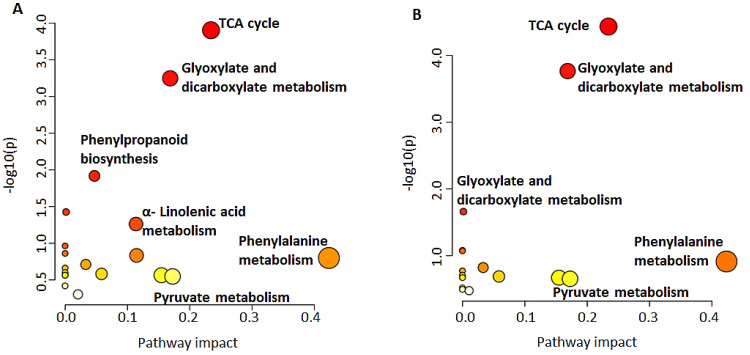
Overview of pathway topology analysis: MetPA-computed metabolic pathways. A graphical depiction of data showing all matched pathways based on *p*-values and pathway impacts. Pathways with low impact to high impact (light yellow to bright red, respectively) active in barley shoots (**A**) and roots (**B**) at 16 days post germination are described according to their significance (pathway impact).

**Figure 7:**
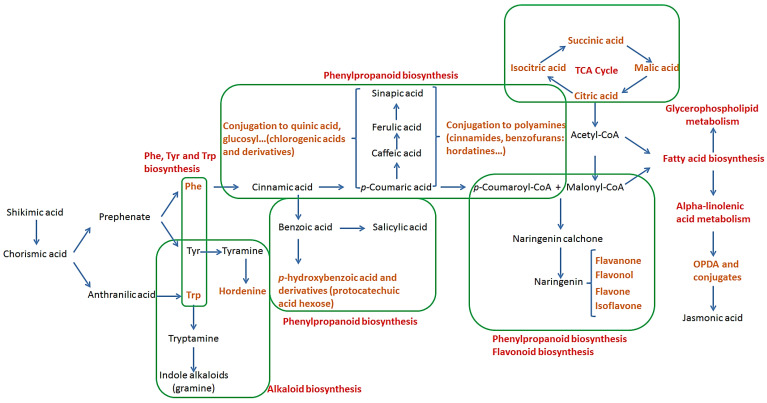
Interlinked pathway summary showing the biosynthesis and participation of all annotated metabolites in barley shoot and root tissues. Annotated metabolites are indicated in orange text, while the general pathway involved is indicated in red and highlighted with green boxes.

Diverse classes of metabolites belonging to both primary and secondary metabolism and synthesised through different metabolic pathways were annotated. The MetPA module of MetaboAnalyst facilitates the pathway network topological analysis and visualisation, as shown in Figure 6. Based on the annotated metabolites (Table 1), pathway analysis with MetPA highlighted 21 metabolic pathways, with 19 in the shoots and 16 in the roots, of which 14 were shared (Table S1). These pathways included tricarboxylic acid (TCA) cycle intermediates, glyoxylate and dicarboxylate metabolism, pyruvate metabolism, α-linolenic acid metabolism, phenylpropanoid biosynthesis as well as phenylalanine, tyrosine and tryptophan biosynthesis. Seven pathways had a significant impact (impact score > 0.10) in the shoots and five had a significant impact in the roots. Several metabolites were not incorporated in these pathways as their database identifiers are currently not available. However, the bioinformatic results highlight their presence in developing barley seedlings and testify to their implication in a large range of biochemical reactions. A summary representation of the biosynthesis of annotated classes of metabolites is shown in Figure 7.

One of the most reported pathways in the biosynthesis of secondary metabolites is that of the phenylpropanoids, originating from the shikimic acid pathway that leads to the formation of chorismic acid. The latter is an intermediate in the biosynthesis of HBAs (e.g., gallic acid and protocatechuic acid) as well as the aromatic amino acids Trp, Tyr and Phe, which are precursors of several secondary metabolites [42]. These amino acids will first undergo deamination, an important step in the formation of phenolic acids. In general, Trp is the precursor of tryptamine and indole-related compounds, such as indole glucosinolates and phytoalexins, as well as indole and quinone alkaloids (not annotated in this study). In the case of Tyr, the transformation into tyramine can lead, after a stepwise *N*-methylation, to the biosynthesis of hordenine, a naturally occurring compound found in diverse plants and mostly reported in germinated barley [43]. Finally, deamination of Phe can result in the formation of cinnamic acid, which is a key molecule in the synthesis of phenolic acids (e.g., HBAs and HCAs) and their derivatives. The esterification of HCAs with quinic acid results in the formation of chlorogenic acids (CGAs) such as those annotated: 3-caffeoylquinic acid and 3- and 4-feruloylquinic acids. They can also be conjugated to polyamines such as agmatine and putrescine to form HCAAs, and, in the case of agmatine, the condensation will lead to different types of hordatines and their glycosylated counterparts. Furthermore, *p*-coumaric acid, together with malonyl-CoA from the TCA cycle, can also undergo a series of reactions resulting in the biosynthesis of different classes of flavonoids, notably flavanones, flavanols and flavones [44,45].

With regard to the fatty acids, de novo biosynthesis mainly occurs in the plastidial compartment, from acetyl CoA, under the coordinated action of acetyl-CoA carboxylase and fatty acid synthase. The resulting octadecanoic acid conjugated to an acyl carrier protein follows the unsaturation program administrated by a sequence of fatty acid desaturases. The formation of polyunsaturated fatty acids (linoleic acid and α-linolenic acid) is associated with that of membrane glycerolipids [46,47,48]. Oxygenation of these fatty acid derivatives can serve as a source of an orchestrated metabolic defence against attack by virulent microorganisms in plants [49]. For example, the jasmonic acid precursor, OPDA, is synthesised either through the linolenic acid or the hexadecatrienoic acid pathway. Jasmonic acid is a polyunsaturated fatty acid-derived molecule capable of undergoing reactions of decarboxylation, hydroxylation, sulphation (12-HSO_4_-JA) and conjugation with amino acids to perform a specific biological function [38].

### 2.3. Metabolite Class Distribution and Roles: Contribution of Barley Hordatines and Biosynthetic Precursors

**Figure 8:**
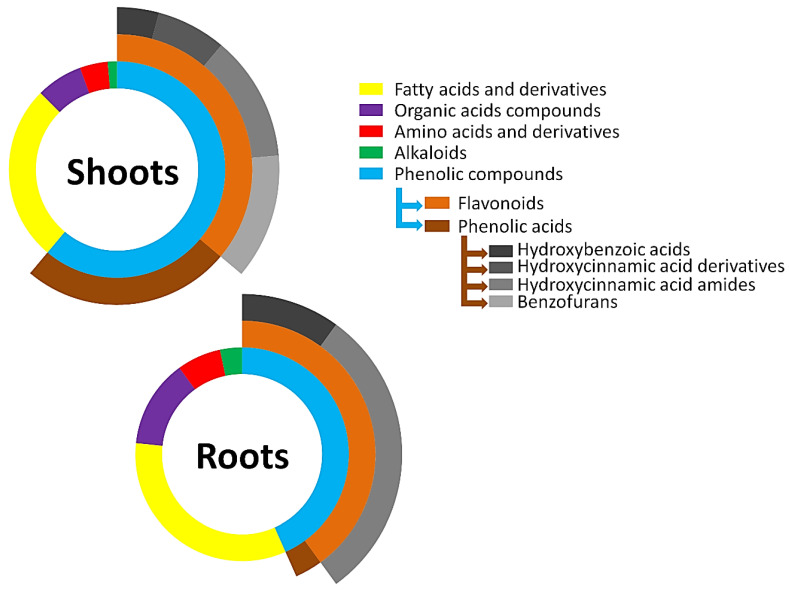
Multi-level 2D doughnut chart showing the classification of metabolites annotated in methanolic extracts from shoot and root tissues of five barley cultivars. The segments are representative of the number of metabolites in the class. The larger the segment, the more metabolites are present in the class. The additional layers represent the subclasses of metabolites present in the phenolic compounds class.

Specialised plant metabolites are unique compounds playing crucial roles in interaction with the environment as well as resilience towards biotic and abiotic stresses. The production of a wide variety of these protective metabolites within a biological system has a significant impact on plant growth and development under various environmental conditions. In general, during germination, biochemical, physiological and morphological changes may occur and affect the survival rate and vegetative growth of seedlings and subsequently have an impact on yield and quality. In this study, environmental conditions were not perturbed, in order that the metabolic profile of the non-stressed barley seedlings could uniquely reflect its early developmental stage (16 d post emergence). Both primary and secondary metabolites were annotated in shoot and root tissues (Table 1; Figure 8).

In general, primary metabolites are highly conserved compounds directly involved in plant growth and development through the mediation of glycolysis and the TCA cycle during photosynthesis [50,51]. Such compounds include lipids or fatty acids as key components of cell membranes and are responsible for a variety of metabolic activities. In addition to providing structural integrity to cells and energy for many metabolic processes, lipids also play an important role as intracellular and extracellular signal transduction mediators [46,52]. Fatty acids, like other primary metabolite classes, have traditionally been assigned purely passive roles in plant defence (e.g., cuticular components). However, recent studies have demonstrated the direct implication of fatty acids and their breakdown products in the activation of diverse plant defence mechanisms. An example is a participation of both C_16_ and C_18_ fatty acids in basal, effector-triggered and systemic immunity [46,49]. In turn, primary metabolites are linked to secondary metabolites by building blocks and biosynthetic enzymes.

**Figure 9:**
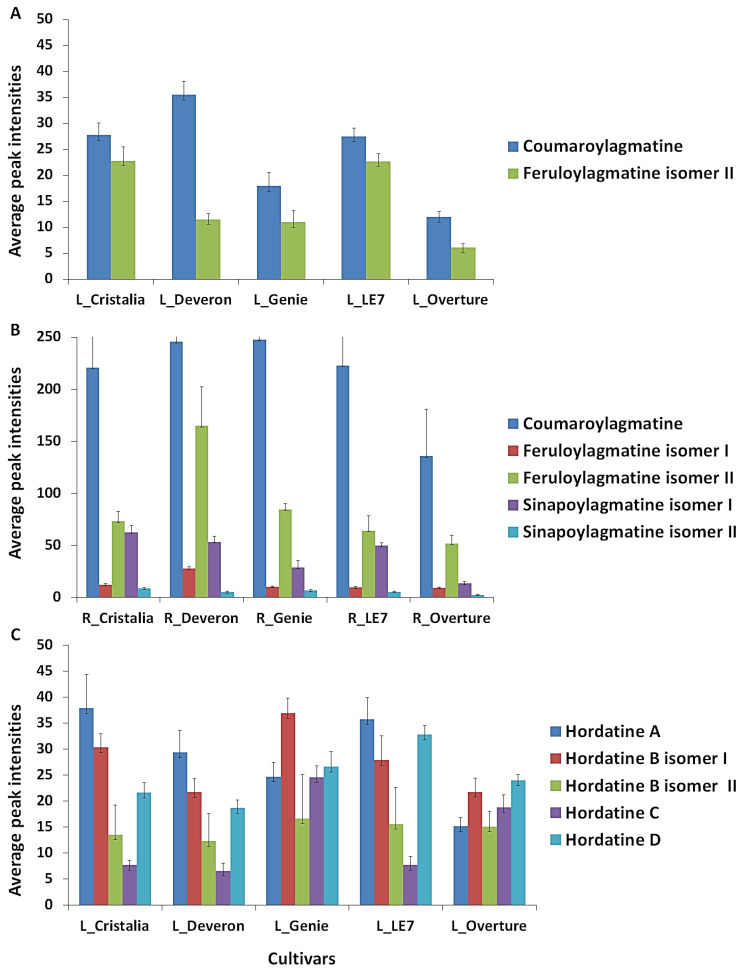
Bar graphs showing the occurrence of barley-specific hordatine metabolites and associated biosynthetic precursors across the cultivars ‘Cristalia’, ‘Deveron’, ‘Genie’, ‘LE7′ and ‘Overture’. (**A**) Hydroxycinnamic acid amides (HCAAs) in the shoot tissue. (**B**) HCAAs in the root tissue. (**C**) Hordatines in the shoot tissue. Each bar is representative of the average peak area corresponding to each metabolite and the error bars indicate standard deviations.

Plants produce a wide range of secondary metabolites, some of which have a prominent role as signalling compounds or in chemical defence against environmental cues. Secondary metabolites are often thought not to be directly involved in primary metabolism; however, though not strictly essential, different studies have now emerged that highlight their significant implications in these processes [53,54,55]. While identifying and annotating metabolites in a biological system is often a challenge in metabolomics studies, understanding the rationale behind their fluctuations in specific conditions as well as exploring their biological activities may be even more difficult. Among the secondary metabolites, phenolic acids, the most abundant phytochemicals annotated in the study (Figure 8), are of substantial morphological and physiological importance in plants. In this category, HCAAs account for the majority and usually play roles in plant growth and development processes. The compounds are also involved in plant defence against diverse environmental stresses [54,55]. *p*-Coumaroyl-, feruloyl- and sinapoyl-substituted amines were the major HCAAs annotated in the study. As seen in Figure 9A,B, their production was cultivar- and tissue-specific. Regardless of the cultivar and the plant tissue, *p*-coumaroylagmatine had the highest relative concentration. It was also noted that the relative abundance of the metabolite was approximately 7–25 times higher in the roots than in the shoots, suggesting a higher activity in the former or that the tissue might be the preferred compartment for the production of the compound.

Although previously reported in roots and shoots, products of the dimerisation of the selected phenolamides, hordatines, were only found in shoot tissue and differentially distributed across cultivars (Table 1; Figure 9C). In ‘Cristalia’, ‘Deveron’ and ‘LE7′, hordatine A was relatively the most abundant and hordatine C the least. By comparison, hordatine B isomer I and hordatine D were more abundant in ‘Genie’ and ‘Overture’, respectively. These observed differences across cultivars highlight a cultivar-specificity in their production and may imply special attributes. Each hordatine may play a specific role in the natural defences of the barley plant. More in-depth analyses of these molecules, separately and across cultivars, are required to reveal their specific involvement in plant growth and development.

Hordatines are important and understudied barley-specific metabolites with reported anti-microbial properties. In vitro studies have revealed that hordatines and *p*-coumaroylagmatine inhibit spore germination in a range of fungal pathogens [56,57,58,59]. Their implication in plant–pathogen interactions has been observed at early growth stages, after germination. Hordatines are also thought to be preformed infection inhibitors (phytoanticipins) because they were identified in significant concentrations in barley young seedlings [59,60,61]. Their precursors, conjugated polyamines, are believed to be implicated in pathogen-induced hypersensitivity responses [10,62] and are more likely constituents of cell walls [63]. To the best of our knowledge, this study is the first to profile and characterise hordatines and other compounds in both shoot and root tissues, at this specific developmental stage in the selected cultivars of barley.

The metabolism of plants is highly compartmentalised; in this regard, understanding the chemical makeup of above- and below-ground tissues of cultivars is critical for identifying common and unique characteristics as well as specific physiological and metabolic roles. Shoots and roots are two autotrophic and heterotrophic functionally and morphologically distinct plant tissues [64], and these functional dissimilarities can be significant contributors to the differential production of chemicals associated with their respective roles. For example, increases in the production of photoprotective compounds such as phenolic acids and flavonoids are observed following strong light exposure. This could explain why shoot extracts included more phenolic compounds than root extracts. Furthermore, differential chemical profiles in shoots vs. roots can add specificity to plant defences against potential shoot or root pathogens in the environment [65].

## 3. Materials and Methods

### 3.1. Barley Plant Material and Cultivation

Barley (*Hordeum vulgare* L.) seeds from two commercial cultivars ‘Overture’ (‘Concerto’ × ‘Quench’) and ‘Cristalia’ (‘Ortolio’ × ‘Brise’) as well as three experimental lines, ‘Deveron’ (‘Genie’ × ‘Tesla’), ‘LE7’ (‘Cristalia’ × ‘Harrington’) and ‘Genie’ (‘NSL04-4299-b’ × ‘Quench’), were provided by the South African Barley Breeding Institute (SABBI, Bredasdorp, South Africa. These cultivars are utilised as irrigated, summer crops grown in the Northern Cape province (Hartswater area) of South Africa.

Prior to cultivation of the five cultivars, the soil (professional germination mix, Culterra, Muldersdrift, South Africa) was pasteurised at 70 °C and seeds were surface-sterilised in 70% ethanol for 5 min before rinsing multiple times with autoclaved distilled water (dH_2_O). Cultivars were grown in a controlled environment and under identical conditions: 12 h dark cycle at temperatures fluctuating between 22–27 °C and 12 h fluorescent light (equivalent to 85 µmol m^−2^·s^−2^). Three independent biological replicates (n = 3) were included in the research. Seedlings were irrigated twice a week; once with dH_2_O and the second time with a water-soluble chemical fertiliser (Multisol ‘N’, Culterra, Muldersdrift, South Africa). Barley shoot and root tissues of each biological replicate were harvested 21 d after planting or 16 d post emergence, when seedlings were at stage 13 according to the Zadoks scale [66]. Root and shoot tissues were separated, snap frozen to quench metabolic activity and stored at −80 °C.

### 3.2. Metabolite Extraction and Sample Preparation

The extraction of each replicate of harvested shoots and roots was performed with 80% cold aqueous methanol (1:10 *w*/*v* ratio). The tissues were homogenised with an Ultra-Turrax homogeniser (CAT, Ballrechten-Dottingen, Germany), and a probe sonicator was used to sonicate for 10 s at 55% power (Bandelin Sonopuls, Berlin, Germany). Homogenates were centrifuged at 5100× *g* for 20 min at 4 °C. The hydro-methanolic supernatants were concentrated to 1 mL using a rotary evaporator (Heidolph Instruments, Schwabach, Germany) and transferred to 2 mL Eppendorf tubes (Eppendorf, Hamburg, Germany) for evaporation at 45 °C in a centrifugal vacuum concentrator to total dryness. Dried extracts were reconstituted in a 1:10 *m/v* ratio with 50% UHPLC-grade methanol by vortexing and sonication (Romil, Cambridge, UK). Prior to ultra-high performance liquid chromatography–quadrupole time-of-flight mass spectrometry (UHPLC–qTOF-MS) analysis, extracts were filtered through 0.22 µm nylon filters into chromatography vials fitted with 500 µL glass inserts, capped and maintained at −20 °C.

### 3.3. Ultra-High Performance Liquid Chromatography (UHPLC)

An Acquity UHPLC system with a photodiode array (PDA) detector coupled to a Waters SYNAPT G1 qTOF mass spectrometer system in V-optics (Waters Corporation, Milford, CT, USA) was used to analyse extracts. The injection volume was 2 µL and extracts were injected onto a Waters HSS T3 C_18_ column (150 mm × 2.1 mm × 1.8 µm), thermostatically controlled at 60 °C. Eluent A and B were, respectively, water and acetonitrile (Romil Pure Chemistry, Cambridge, UK) and both contained 0.1% formic acid. The concave gradient elution used a binary solvent at a flow rate of 0.4 mL·min^−1^. The overall run duration was 30 min, and each sample was examined three times. The elution began with 5% B for 1 min and gradually increased to 95% B over the course of 24 min. For 2 min, the concentration of B remained constant at 95% before returning to the initial settings at 27 min. The analytical column was allowed to re-equilibrate for 3 min before the next injection. In order to reduce measurement bias, all sample vials were randomised. The LC-MS system was assessed with quality control (QC) samples (consisting of representative pooled samples), and the background noise level as well as carry-over were monitored with the integration of blanks (50% MeOH). Each sample was analysed in triplicate.

### 3.4. High-Definition Mass Spectrometry

The ionisation of the analytes was achieved using electrospray ionisation (ESI) in both positive and negative modes. The MS parameters were as follows: the capillary, sampling and extraction cone voltages were 2.5 kV, 40 V and 4.0 V, respectively; the desolvation temperature was set at 450 °C while the source temperature was set at 120 °C; the gas fluxes for the cone and desolvation were 50 L.h^−1^ and 550 L.h^−1^, respectively. A mass range of 50 to 1200 *m*/*z* was selected with a scan time of 0.1 s. Nitrogen was used as the nebulization gas at a flow rate of 700 L.h^−1^. The reference calibrant, leucine encephalin (50 pg·mL^−1^, [M + H]^+^ = 556.2771 and [M − H]^−^ = 554.2615), was continuously sampled every 15 sec with a lock mass flow rate of 0.1 mL·min^−1^, producing an average intensity of 350 counts/scan in centroid mode. The reference enabled the processing software (MassLynx^TM^, Waters Corporation, Milford, MA, USA) to automatically adjust for small deviations in centroid masses found in the sample. This yielded a mass accuracy window of 0.5 Da and typical mass accuracy of 1 to 3 mDa. Different collision energies (MS^E^, 0–40 eV) were applied to generate fragmentation data and extract the structural information of detected metabolites. Three analytical/technical replicates were created by analysing each of the three biological replicates in triplicate. As a result, nine data points per condition (n = 3 × 3 = 9) were generated, fulfilling the requirement for processing by multivariate data analysis (MVDA).

### 3.5. Data Pre-Processing, Multivariate Data Analyses and Statistical Modeling

MassLynx XS^TM^ 4.1 software (Waters Corporation, Manchester, UK) was used to visualise and process the raw UHPLC-MS data. The specifications of the MarkerLynx XS^TM^ application, which is part of the Masslynx XS^TM^ software, were set to analyse mass chromatograms within the retention time (Rt) range of 0.6 to 25 min, a mass range of 50 to 1200 Da and a mass tolerance of 0.05 Da. Peak alignment across samples was performed within the range of ±0.05 Da for the masses and ±0.20 min for the Rt window. Therefrom, a matrix output was obtained and consisted of Rt-*m*/*z* variable pairs as well as the corresponding peak area for each sample. For multivariate data analysis and modelling, the generated matrices were exported to the soft independent modelling of class analogy (SIMCA) software version 15, containing the ‘omics’ skin (Sartorius, Stedim Data Analytics AB, Umeå, Sweden). Only matrices with a noise level below 50% were used.

The data obtained were *Pareto*-scaled to maintain the data structure closed to the original, to rectify measurement errors, to balance all variables and reduce redundancy. As a means to reduce the dimensionality and explore the data, the unsupervised chemometric methods, principal component analysis (PCA) and hierarchical cluster analysis (HiCA), were performed. A nonlinear iterative partial least squares algorithm (in-built within the SIMCA software) was used to handle the missing values. A seven-fold cross-validation (CV) was applied in computing the chemometric models, and the PCA models generated were assessed with model diagnostic tools, i.e., (R^2^X), the ‘goodness of fit’ parameter/the explained variation parameter, and (Q^2^), the ‘goodness of prediction’ ability/predicted variation parameter. The closer these diagnostic parameters were to 1.0, the more robust and valid were the models. The HiCA algorithm clustered observations in an agglomerative manner, based on their differences and similarities to provide more defined subclustering.

### 3.6. Metabolite Annotation

The assignment of the structural identity of the acquired mass spectral information is a crucial step in metabolomics studies. This determines or impacts the understanding of biological processes occurring in the plant at a specific time point and under well-defined environmental conditions. This was accomplished through the comparison of the acquired mass spectral information that included the described MS^E^ method (data-independent acquisition, DIA) where the MS analyses were performed using non-fragmented as well as five fragmenting experiments concurrently, by applying alternating collision energy of 3 eV (unfragmented) and from 10 to 40 eV (fragmented). Accurate mass and mass fragmentation data were compared with those in libraries and databases, such as PubChem [67,68], Massbank of North America [67], MS-Dial [69] ChemSpider [70] (http://www.chemspider.com/, accessed on 21 January 2022), Lipidmaps [71] and the Dictionary of Natural Products [72] as well as data reported in the published literature. The papers used for this purpose are cited in the results section. All metabolites were annotated following the Metabolomic Standards Initiative (MSI) levels 2 and 3. Hordenine (N,N-dimethyltyramine, a phenethylamine alkaloid; MW 165.23 g·mol^−1^) was confirmed with an authentic standard (Sigma-Aldrich, Muenchen, Germany).

### 3.7. Metabolomics Pathway Analysis

Data from the annotated metabolites, such as metabolite identity, the relationships between metabolites and changes in levels, were used to build global metabolic interrelationships such as metabolic pathway mapping. In the study, Metabolomics Pathway Analysis (MetPA), an integral component of the MetaboAnalyst bioinformatics tool package (version 5.0) [73], was used to identify significant metabolic pathways defining the early developmental (post-germination) growth stage of barley. MetPA uses multiple pathway enrichment analysis methodologies as well as the analysis of pathway topological properties to facilitate the identification of the most significant metabolic pathways involved in a given metabolic investigation. The identities of the annotated metabolites, assigned as KEGG (Kyoto Encyclopedia of Genes and Genomes) identifiers [74], were uploaded into the MetPA tool for pathway topological analysis, which uses a ‘relative betweenness centrality’ parameter and a hypergeometric test algorithm to simplify pathway network topological analysis and visualisation.

## 4. Conclusions

The contribution of metabolomics to plant science research has increased considerably in recent years and has led to its application in different agricultural sectors, including plant breeding and research related to the metabolic basis of disease resistance. This research includes studies on cultivars differing in their performance attributes, such as the spectrum of disease resistance or susceptibility, where the occurrence of signatory metabolites can be used for explorative analysis in forward genetics approaches to discover genes associated with a resistant phenotype. Besides the complexity of the task of measuring a biological system’s entire range of metabolites, one of the most difficult aspects of metabolomic research is the identification of relevant metabolites and the determination of their biological activities. Investigating plant cultivars or breeding lines at an early developmental stage and when not subjected to external stress stimuli provides more information on the innate capabilities and potential of natural defences. Metabolomics profiling of shoots and roots of five barley cultivars revealed both primary and secondary metabolites present at a young developmental stage. Given the absence of stressors, the identified metabolites could be associated with the plants’ growth and developmental processes as well as the natural phytochemical defences they contain. Among these metabolites, the occurrence of an important class of multifunctional compounds (HCAAs, including hordatines and precursors) was highlighted, and their relative distribution across different tissues and cultivars indicates the specificity in the metabolite production. In fact, *p*-coumaroylagmatine, the primary substrate in the biosynthesis of hordatine A and B, was significantly higher in the roots as compared to the shoots. On the other hand, the hordatines were only found in the shoot and not in the roots. Plants have highly compartmentalised metabolic networks, and the biochemical steps of a single pathway may take place in different locations. The hordatines and associated precursors and derivatives could thus serve as potential markers linked to desired performance traits in crop improvement practices, e.g., to obtain enhanced disease-resistance capabilities. Considering the number of phenolic compounds annotated in the study, the phenylpropanoid pathway involved in their biosynthesis appears to be very active at this specific growth stage. Combined with chemometric tools and modelling, the selection of metabolic features that contribute to the characteristic properties of specific cultivars can be achieved. The integration of targeted metabolomics profiling may thus allow further assessment of the quantitative aspects of the metabolome phenotype of a given system. Metabolite profiling can be performed to assist with the selection process during plant breeding workflows but also to evaluate or assess a newly bred cultivar for the presence of signatory markers of desired traits.

## Figures and Tables

**Table 1:** List of annotated metabolites extracted from shoots and roots of the barley cultivars ‘Overture’, ‘Cristalia’, ‘Deveron’, ‘LE7′ and ‘Genie’ from the Northern Cape region of South Africa. Metabolites were annotated to the Metabolomics Standards Initiative, level 2 (tentative identification).

No	Compounds	Rt (min)	*m*/*z* (ESI–)	Diagnostic Fragments	*m*/*z* (ESI+)	Diagnostic Fragments	Shoots	Roots
	**Hydroxybenzoic acids**							
1	Protocatechuic acid hexose	1.69	315.0749	153			√	√
2	Benzylalcohol-hexose-pentose	5.66	401.1405	401, 269, 161, 101			√	√
3	Gallic acid monohydrate	12.56	187.0942	169, 125			√	√
	**Hydroxycinnamic acids and derivatives**							
4	Ferulic acid hexose	1.76	355.0676	193			√	
5	3-Caffeoylquinic acid	2.16	353.0865	191, 179			√	
6	3-Feruloylquinic acid	4.08	367.1053	367, 193, 134			√	
7	Sinapic acid hexose	5.37	385.1129	385, 223, 164			√	
8	4-Feruloyquinic acid	7.44	367.0996	367, 193, 173			√	
	**Hydroxycinnamic acid amides**							
9	*p*-Coumaroylhydroxyagmatine hexoside	1.82			455.2128	293, 147	√	
10	*p*-Coumaroylputrescine	2.46			235.1346	218, 188, 147, 119, 91		√
11	Feruloylhydroxyagmatine hexoside	2.57			485.2265	323, 177	√	
12	*p*-Coumaroylhydroxyagmatine	2.66	291.1426	119	293.1544	147	√	√
13	*p*-Coumaroylagmatine hexoside	2.85			439.2234	277, 217, 147, 119	√	
14	Feruloylagmatine isomer I	3.29			307.1745	307, 177, 147/145		√
15	Feruloylhydroxyagmatine	3.54			323.1679	323, 177	√	√
16	*p*-Coumaroylagmatine	4.23			277.1581	277, 217, 147, 131	√	√
17	Feruloylagmatine hexoside	4.45			469.2355	307, 293, 177, 147, 119	√	
18	Feruloylagmatine isomer II	5.49			307.1705	307, 177, 145	√	√
19	Sinapoylagmatine isomer I	6.41			337.1794	337, 207, 175, 147, 119		√
20	Sinapoylhydroxyagmatine	6.54	351.1222	351, 249, 101			√	√
21	Sinapoylagmatine isomer II	7.89			337.1819	337, 207, 175, 147, 120		√
	**Benzofurans**							
22	Hordatine B isomer I	7.60	579.2993	579, 423, 267	581.3153	581, 425, 321, 295, 293, 157, 131, 114	√	
23	Hordatine B isomer II	7.72			581.3143	581, 425,321, 291, 157, 131, 114	√	
24	Hordatine D	7.82			641.1716	641, 425, 291, 157, 131, 141, 118	√	
25	Hordatine A	7.93	549.2915	549, 393, 385, 267, 249	551.3013	551, 425, 395, 291, 276, 265, 157, 131, 114	√	
26	Hordatine C	8.25	609.3073	579, 453, 423, 393, 297, 237	611.3342	611, 581, 455, 425, 395, 325, 306, 157, 131, 114	√	
27	Hordatine C hexose isomer I	3.56	771.2131	771, 609, 593, 503, 473			√	
28	Hordatine B hexose	4.03	787.3706	787 (741 + formic acid), 741, 579	743.382	743, 564, 425, 372, 291, 261, 157	√	
29	Hordatine A hexose	4.34	757.3595	757 (711 + formic acid), 711, 549, 393, 131	713.3724	713, 551, 533, 395, 357, 276, 247, 131	√	
30	Hordatine C hexose isomer II	5.41	771.1981	771, 609, 467, 205, 190			√	
	**Flavonoids**							
31	Isoorientin-7-*O*-glucoside/Lutonarin	6.74	609.1421	609, 447, 327	611.1583	611, 449, 431, 383, 353, 329, 299, 329	√	
32	Isovitexin-7-*O*-glucoside/Saponarin	8.64	593.1534	593, 473, 431, 341, 311			√	√
33	Isovitexin-7-*O*-rhamnosyl-glucoside	9.04	739.2300	739, 431, 341, 311			√	
34	Isoscoparin-7-*O*-glucoside	9.20	623.1533	623, 461, 341			√	
35	Isovitexin-7-*O*-[6″-sinapoyl]-glucoside 4′-*O*-glucoside	9.40	961.2755	961, 799, 593, 431, 311			√	
36	Isovitexin derivative	9.91	611.2522	611, 431, 251, 207			√	
37	Isovitexin 2″-*O*-glucoside	10.01	593.1427	593, 413, 293	595.1627	595, 433, 415, 367, 337, 283	√	
38	Isovitexin 2″-*O*-arabinoside	10.15	563.134	563, 413, 293	565.1579		√	
39	Luteolin 7-*O*-arabinosylglucoside	10.91	579.1371	579, 447, 285			√	
40	Isovitexin-7-*O*-[6″-sinapoyl]-glucoside	11.59	799.2158	431, 341, 311	801.2339	801, 783, 747, 681, 621, 397, 379, 283	√	
41	Isoscoparin-7-*O*-[6″-sinapoyl]-glucoside	11.69	829.2297	829, 461, 341			√	
42	Isovitexin-7-*O*-[X″-feruloyl]-glucoside	11.98	769.2041	769, 431, 311	771.2312	771, 415, 379, 361, 313, 283, 177	√	
43	Apigenin-7-*O*-arabinosylglucoside	12.06	563.14	563, 269			√	
44	Isoscoparin-7-*O*-[6″-feruloyl]-glucoside	12.10	799.2159	799, 461, 341			√	
45	Chrysoeriol-7-*O*-arabinosyl-glucoside	12.37	593.144	593, 299	595.1630	595, 463, 301, 262	√	
46	6-Prenylnaringenin	19.18	339.2123	339, 307, 321, 289			√	
47	Isoorientin-7-*O*-[6″-sinapoyl]-glucoside	10.71	815.2056	815, 447, 327			√	
48	Flavonoid-related compound	11.03			787.2163	431, 413, 395, 383, 377, 365, 353, 329, 299,177	√	
	**Alkaloids**							
49	Hordenine	1.17			166.1139	121	√	√
	**Amino acids and derivatives**							
50	Phenylalanine	1.68	164.0699	147, 101	166.0823	120, 103, 93, 91	√	√
51	Tryptophan	2.51	203.0767	116, 142, 158/159, 203	205.0928	188, 146, 118, 91	√	√
52	N-Acetylaspartylglutamic acid	6.03	303.0826	96			√	
	**Organic acids compounds**							
53	Isocitric acid	0.92	191.0038	111			√	√
54	Malic acid	1.02	133.0120	115			√	√
55	Citric acid	1.16	191.0066	111, 173			√	√
56	Succinic acid	1.20	117.0103	117			√	√
57	Citric acid derivative	1.41	306.1123	191, 173, 111			√	
	**Fatty acids and derivatives**							
58	12-hydroxyjasmonate sulfate	4.59	305.0635	305, 225, 96			√	
59	α-Linolenoyl ethanolamide	9.87			322.2772			√
60	Linoleoyl ethanolamide	12.33			324.2901			√
61	(10E,15Z)-9,12,13-trihydroxyoctadeca-10,15-dienoic acid isomer I (9,12,13-TriHODE)	16.57	327.2131	327, 229, 211, 171, 113			√	
62	9,12,13-TriHODE isomer II	16.67	327.2170	327, 229, 211			√	√
63	9,12,13-TriHODE isomer III	16.79	327.2132	327, 229, 211			√	
64	Trihydroxyoctadecenoic acid	17.38	329.2278	329, 229, 211			√	√
65	9-Oxo-12,13-dihydroxy-10E,15Z-octadecadienoic acid (9K,12,13-diHODE)	17.61	325.1967	325, 307, 209			√	√
66	OPDA conjugate isomer I	19.61	309.2024	309, 291, 273, 247			√	√
67	OPDA conjugate isomer II	19.68	309.1991	309, 291, 273, 247, 209, 179, 165			√	√
68	9-Hydroxy-12-oxo-10(E),15(Z)-octadecadienoic acid isomer I (12K, 9-HODE)	20.09	309.2034	309, 291, 197			√	
69	12K, 9-HODE isomer II	20.59	309.2019	309, 291, 247, 165			√	√
70	Linolenic acid derivative I, isomer I	20.77	675.3553	675, 415, 397, 277, 235, 89			√	
71	12-Oxo-phytodienoic acid (12-OPDA)	21.11	291.1946	291, 273, 247, 165			√	
72	Hydroxyoctadecadienoic acid/hydroxylinoleic acid	22.37	295.2256	277, 233, 195			√	√
73	Linolenic acid derivative I, isomer II	21.07	675.3615	675, 415, 397, 277, 235, 89			√	
74	Linolenoylglycerol/monolinolenin isomer I	20.81			353.2632	353, 335, 261, 243	√	√
75	Linolenoylglycerol/monolinolenin isomer II	21.11			353.2595	353, 335, 261, 243	√	
76	Linolenoylglycerol/monolinolenin isomer III	21.27			353.2644	353, 331, 261, 243	√	
77	Linolenoylglycerol/monolinolenin isomer VI	21.94			353.2625	353, 331, 261, 243	√	
78	Linolenic acid derivative II	22.69	445.2328	445, 311, 293, 277			√	

√ = indicates presence in tissue type (shoots vs. roots).

## Data Availability

The study design information, LC-MS data, data processing and analyses are reported and incorporated in the main text. Raw data, analyses and data processing information and the meta-data are being deposited in the EMBL-EBI metabolomics repository—MetaboLights50, with the identifier MTBLS3142, (http://www.ebi.ac.uk/metabolights/MTBLS3142 accessed on 10 March 2022).

## Supplementary Materials

The following supporting information can be downloaded at: https://www.mdpi.com/article/10.3390/metabo12040310/s1, Figure S1: Ultra-high performance liquid chromatography mass spectrometry (UHPLC-MS) base peak intensity (BPI) chromatograms in negative electrospray ionisation (ESI) mode of (A) leaf and (B) root extracts from five different barley cultivars (‘Overture’, ‘Cristalia’, ‘Deveron’, ‘LE7′ and ‘Genie’) under controlled conditions and harvested 16 days post germination, Figure S2: Ultra-high performance liquid chromatography mass spectrometry (UHPLC-MS) base peak intensity (BPI) chromatograms in positive electrospray ionisation (ESI) mode of (A) leaf and (B) root extracts from five different barley cultivars (‘Overture’, ‘Cristalia’, ‘Deveron’, ‘LE7′ and ‘Genie’) under controlled conditions and harvested 16 days post germination, Figure S3: Principal component analysis (PCA) score plot models and hierarchical clustering analyses (HiCAs) for shoot and root tissues of five cultivars of *Hordeum vulgare* (Northern Cape region of South Africa) based on ESI(+) data, Table S1: Significant metabolic pathways active in barley shoots and roots (at 16 days post emergence), based on selected annotated metabolites in methanolic extracts.
