# Supplementary material for: Hordatines and Associated Precursors Dominate Metabolite Profiles of Barley (Hordeum vulgare L.) Seedlings: A Metabolomics Study of Five Cultivars
Source: Metabolites. 2022 Mar 31;12(4):310. doi: 10.3390/metabo12040310 (PMC9030721; doi:10.3390/metabo12040310)
Supplement: Supplementary file 1 [file metabolites-12-00310-s001.zip › metabolites-1657989-supplementary.pdf]

metabolites

MDPI

Supplementary File

Hordatines and associated precursors dominate metabolite profiles of barley (*Hordeum vulgare* L.) seedlings: A metabolomics study of five cultivars

Claude Y Hamany-Djande, Paul A. Steenkamp, Lizelle A. Piater, Fidele Tugizimana and Ian A. Dubery*.

Research Centre for Plant Metabolomics, Department of Biochemistry, University of Johannesburg, P.O. Box 524, Auckland Park, Johannesburg 2006, South Africa; 201410297@student.uj.ac.za (C.Y.H-D.); ftugizimana@uj.ac.za (F.T.); psteenkamp@uj.ac.za (P.A.S.); lpiater@uj.ac.za (L.A.P.)

* Correspondence: idubery@uj.ac.za (I.A.D.); Tel.: +27-11-5592401

Figure S1. Ultra-high performance liquid chromatography - mass spectrometry (UHPLC-MS) base peak intensity (BPI) chromatograms in negative electrospray ionisation (ESI) mode of (**A**) leaf and (**B**) root extracts from five different barley cultivars ('Overture', 'Cristalia', 'Deveron', 'LE7' and 'Genie') under controlled conditions and harvested 16 days post-germination.

Figure S2. Ultra-high performance liquid chromatography - mass spectrometry (UHPLC-MS) base peak intensity (BPI) chromatograms in positive electrospray ionisation (ESI) mode of (**A**) leaf and (**B**) root extracts from five different barley cultivars ('Overture', 'Cristalia', 'Deveron', 'LE7' and 'Genie') under controlled conditions and harvested 16 days post-germination.

Figure S3. Principal components analysis (PCA) score plot models and hierarchical clustering analyses (HiCA) for shoot and root tissues of five cultivars of *Hordeum vulgare* (Northern Cape region of South Africa) based on ESI(+) data.

Table S1. Significant metabolic pathways active in barley shoots and roots (at 16 days post emergence), based on selected annotated metabolites in methanolic extracts.

metabolites

MDPI

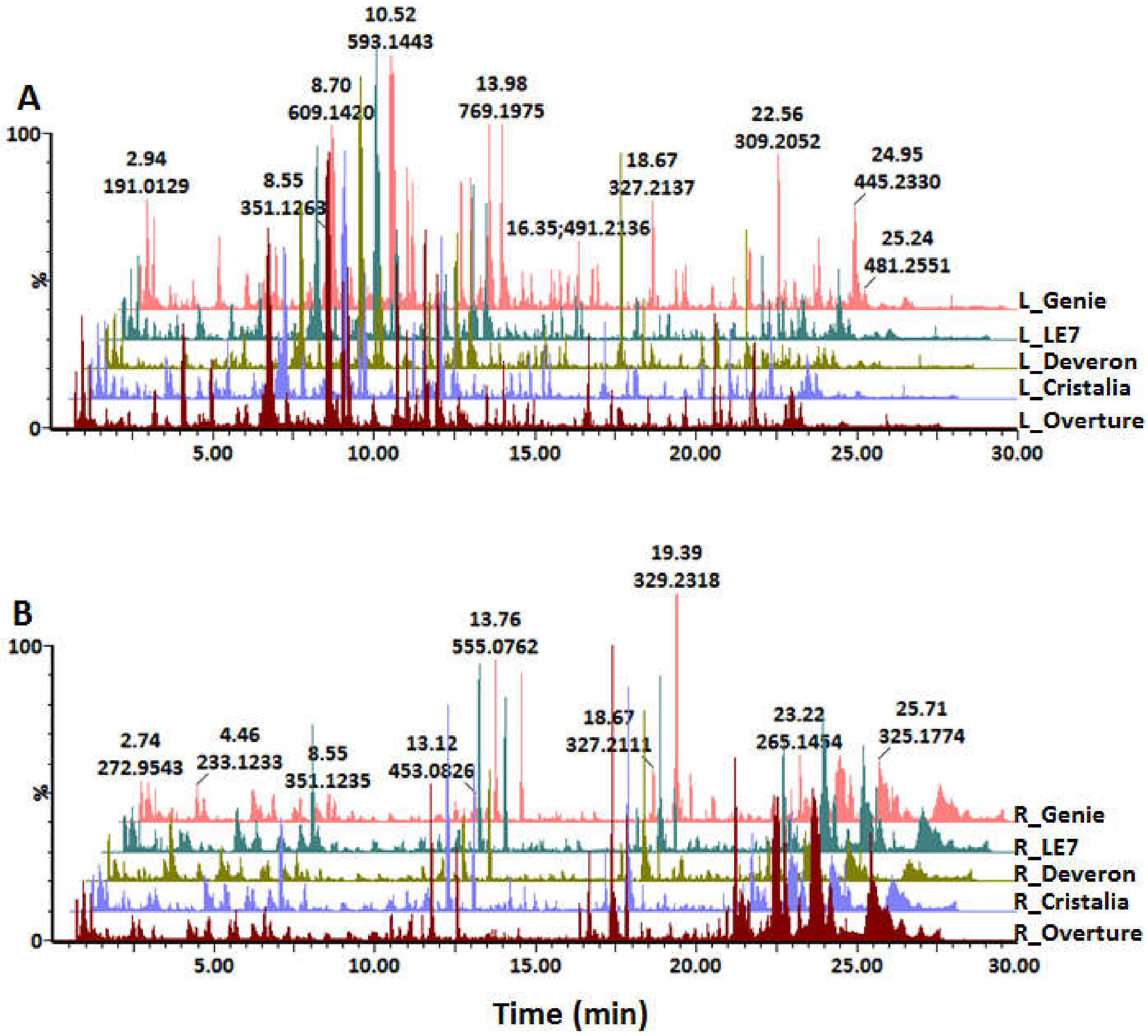

Figure S1. Ultra-high performance liquid chromatography - mass spectrometry (UHPLC-MS) base peak intensity (BPI) chromatograms in negative electrospray ionisation (ESI) mode of (**A**) leaf and (**B**) root extracts from five different barley cultivars (‘Overture’, ‘Cristalia’, ‘Deveron’, ‘LE7’ and ‘Genie’) under controlled conditions and harvested after 21 days.

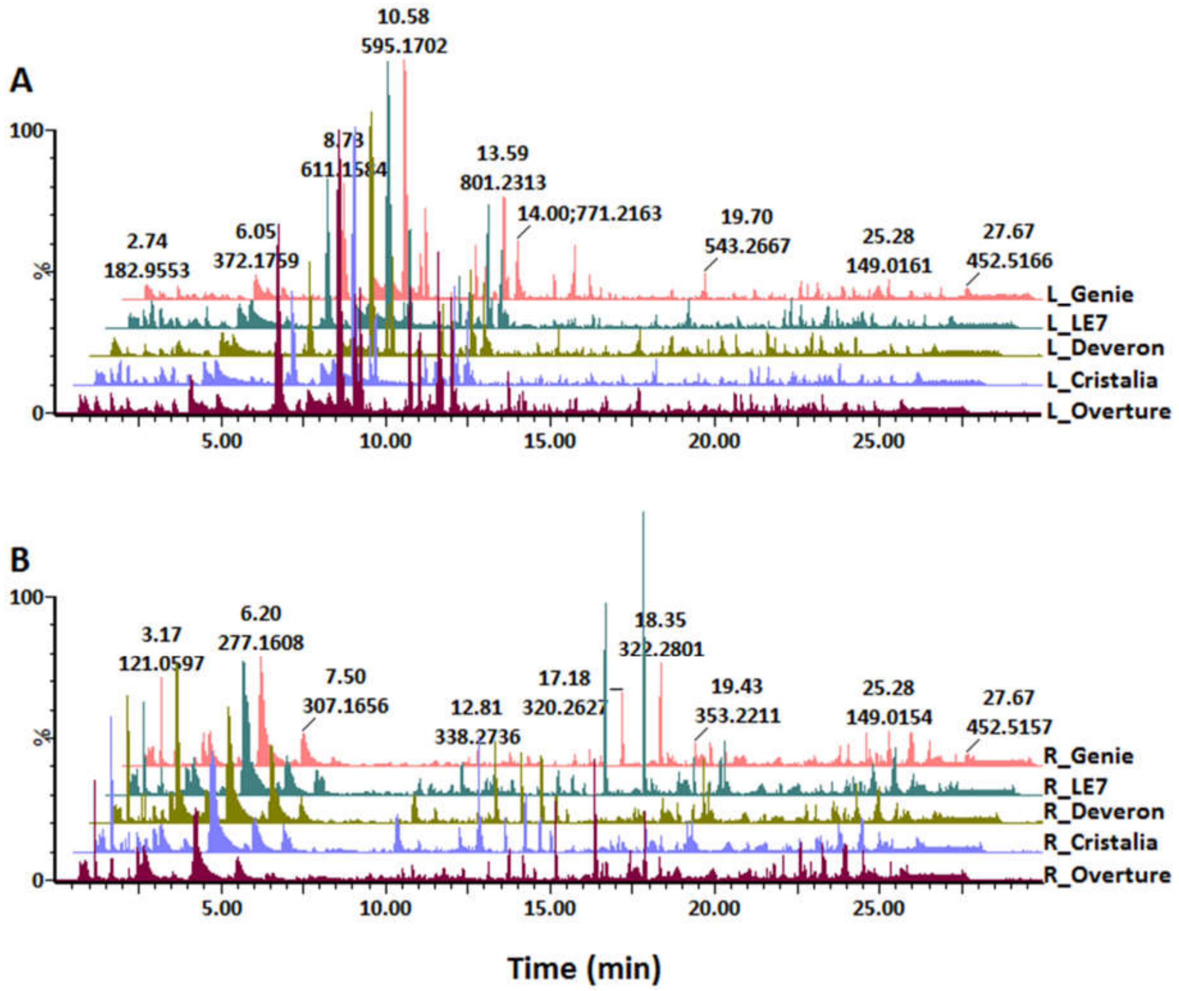

Figure S2. Ultra-high performance liquid chromatography - mass spectrometry (UHPLC-MS) base peak intensity (BPI) chromatograms in positive electrospray ionisation (ESI) mode of (**A**) leaf and (**B)** root extracts from five different barley cultivars ('Overture', 'Cristalia', 'Deveron', 'LE7' and 'Genie') under controlled conditions and harvested after 21 days.

metabolites

MDPI

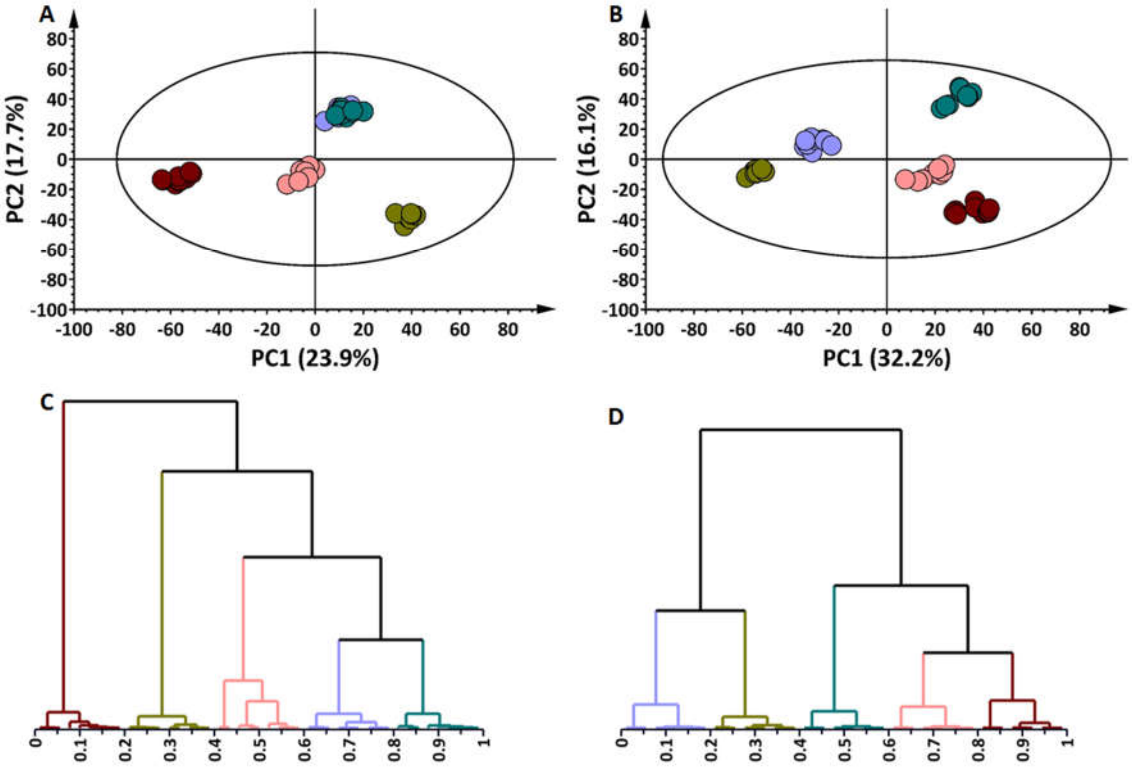

Figure S3. Principal components analysis (PCA) score plot models and hierarchical clustering analyses (HiCA) for shoot and root tissues of five cultivars of *Hordeum vulgare* (Northern Cape region of South Africa). The calculated Hoteling's T2 with a 95% confidence interval is represented by the ellipses present in each PCA model. The PCA plots illustrate the similarities or differences within (intra-cultivar variance by PC2) and between (inter-cultivar variance by PC1). (**A**): Shoot tissue: 6-component model explaining 64.9% variation and predicting 47.7% variation, (**B**): Root tissue: 5-component model explaining 76.4% variation and predicting 67.2% variation. (**C**): HiCA dendrogram showing the hierarchical structure of shoot data and corresponding to the PCA model in (**A**); and (**D**): HiCA dendrogram showing the hierarchical structure of root data and corresponding to the PCA model in (**B**). Data was acquired from hydromethanolic extracts, analysed by UHPLC–qTOF–MS in ESI(+) mode.

Table S1. Significant metabolic pathways active in barley shoots and roots (at 16 days post emergence), based on selected annotated metabolites in methanolic extracts. The list was generated from Metabolomics Pathway Analysis (MetPA) in MetaboAnalyst 5.0.

		**Shoots**		**Roots**	
No	**Pathway Names**	**Impact**	**Significance (*p*-values)**	**Impact**	**Significance (*p*-values)**
1	Phenylalanine metabolism	0.42308	0.15884	0.42308	0.12146
2	Citrate cycle (TCA cycle)	0.23397	1.26E-04	0.23397	3.72E-05
3	Tryptophan metabolism	0.17241	0.28313	0.17241	0.22057
4	Glyoxylate and dicarboxylate metabolism	0.16853	3.2452	0.16853	3.7641
5	Pyruvate metabolism	0.15462	0.2726	0.15462	0.21201
6	Stilbenoid, diarylheptanoid and gingerol biosynthesis	0.11458	0.14658	-	-
7	alpha-Linolenic acid metabolism	0.11368	0.054769	-	-
8	Carbon fixation in photosynthetic organisms	0.05846	0.26192	0.05846	0.20337
9	Phenylpropanoid biosynthesis	0.0470	0.012126	0	0.31674
10	Sulfur metabolism	0.03315	0.19463	0.03315	0.1496
11	Flavonoid biosynthesis	0.02065	0.30406		
12	Glycerophospholipid metabolism	-	-	0.0107	0.47933
13	Phenylalanine, tyrosine and tryptophan biosynthesis	0.0015	0.037608	0.0015	0.02179
14	Alanine, aspartate and glutamate metabolism	0	0.2726	0	0.21201
15	Aminoacyl-tRNA biosynthesis	0	0.13762	0	0.084234
16	Biosynthesis of unsaturated fatty acids	0	0.2726	-	-
17	Butanoate metabolism	0	0.21768	-	-
18	Butanoate metabolism	-	-	0	0.16789
19	Glycine, serine and threonine metabolism	0	0.3808	0	0.30152
20	Propanoate metabolism	0	0.25109	0	0.19463
21	Tropane, piperidine and pyridine alkaloid biosynthesis	0	0.10877	0	0.082597

(-) Not present in the corresponding plant tissue.
